# Insights and perspectives on the enigmatic alary muscles of arthropods

**DOI:** 10.3389/fcell.2023.1337708

**Published:** 2024-01-15

**Authors:** Laetitia Bataillé, Gaëlle Lebreton, Hadi Boukhatmi, Alain Vincent

**Affiliations:** ^1^ Molecular and Developmental Biology Unit (MCD), Centre de Biologie Intégrative (CBI), Université de Toulouse, CNRS UMR 5077, Toulouse, France

**Keywords:** alary muscles, arthropods, body architecture, circulatory system, metamorphosis, trans-differentiation

## Abstract

Three types of muscles, cardiac, smooth and skeletal muscles are classically distinguished in eubilaterian animals. The skeletal, striated muscles are innervated multinucleated syncytia, which, together with bones and tendons, carry out voluntary and reflex body movements. Alary muscles (AMs) are another type of striated syncytial muscles, which connect the exoskeleton to the heart in adult arthropods and were proposed to control hemolymph flux. Developmental studies in *Drosophila* showed that larval AMs are specified in embryos under control of conserved myogenic transcription factors and interact with excretory, respiratory and hematopoietic tissues in addition to the heart. They also revealed the existence of thoracic AMs (TARMs) connecting to specific gut regions. Their asymmetric attachment sites, deformation properties in crawling larvae and ablation-induced phenotypes, suggest that AMs and TARMs could play both architectural and signalling functions. During metamorphosis, and heart remodelling, some AMs trans-differentiate into another type of muscles. Remaining critical questions include the enigmatic modes and roles of AM innervation, mechanical properties of AMs and TARMS and their evolutionary origin. The purpose of this review is to consolidate facts and hypotheses surrounding AMs/TARMs and underscore the need for further detailed investigation into these atypical muscles.

## Introduction

**FIGURE 1:**
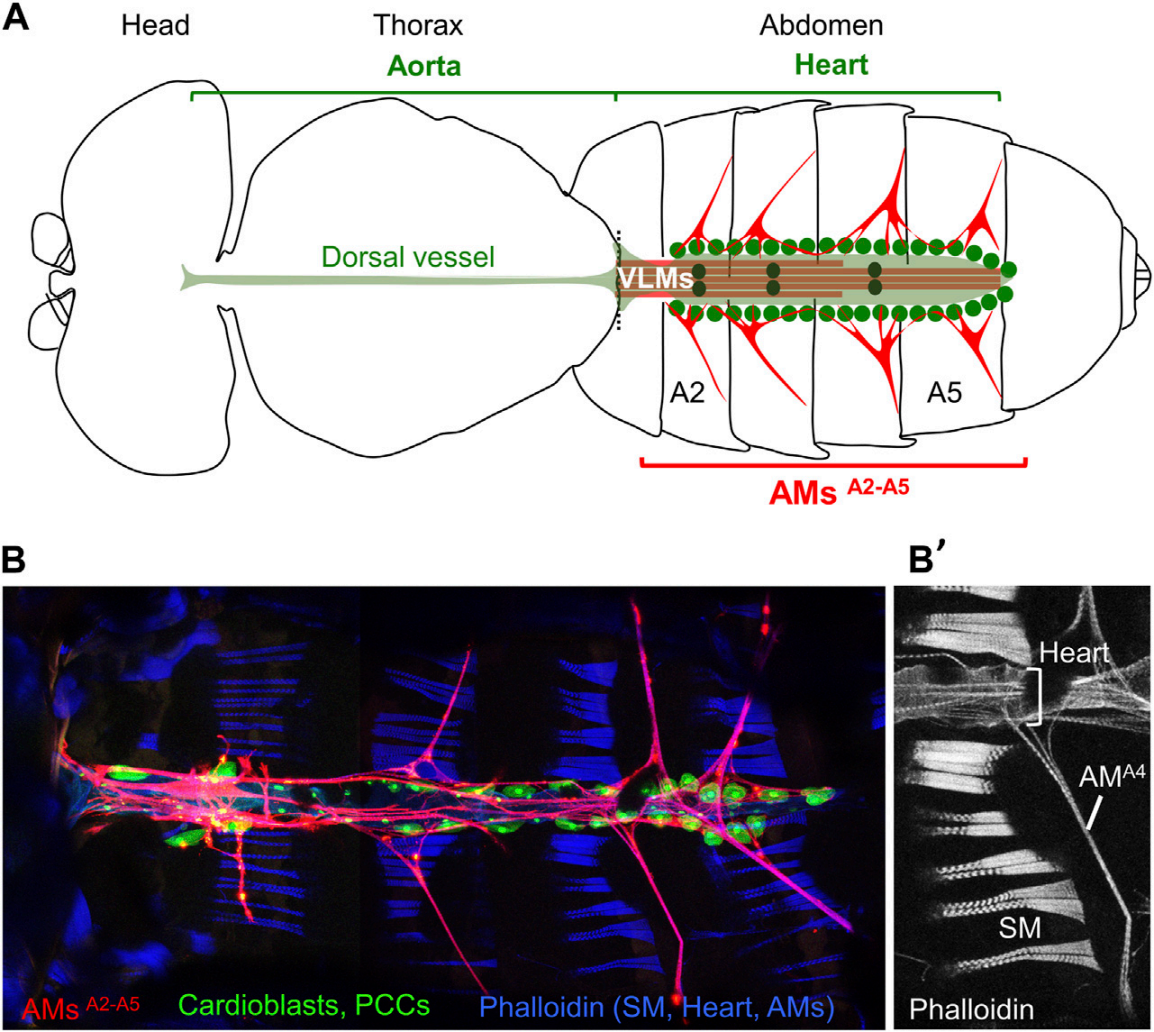
*Drosophila* adult dorsal vessel and AMs. **(A)** Schematic drawing of an adult *Drosophila* (modified from Miller, A., 1950). Alary Muscles (AMs) in abdominal segments A2 to A5 (AMs^A2-A5^) are drawn in red. The ventral longitudinal muscle (VLM, brown) is located underneath the heart (pale green). Green dots indicate pericardial cells, and black dots the 3 pairs of valve cells. **(B)** Confocal view of the heart and AMs. Dissected *AME*_*R*_ *-Gal4; UAS-cd4-tdTomato, HandC-GFP* adult (Bataillé et al., 2020) stained with Phalloidin, showing AMs in red, pericardial cells (PCCs) and cardiomyocytes in green, and dorsal abdominal skeletal muscles (SM) in blue. **(B’)** Phalloidin staining of the A4 segment, dorsal Z sections, showing the SMs, heart and AM^A4^.

Alary muscles (AMs) were identified from anatomical studies of the circulatory system in the abdomen of adult arthropods and described as multinucleated striated myofibers connecting the heart to the lateral exoskeleton (Miller, 1950; De Wilde, 1948; Alexandrowicz, 1954; Jones, 1954; Adams et al., 1973; Table 1). AMs take their name from their wing (*alae*)-like shape and are sometimes termed suspensory ligaments or alary ligaments in crustacea. In adult insects, the circulatory system, called dorsal vessel, extends from the head to the abdomen and is responsible for the intracelomic flux of hemolymph. It is located medio-dorsally in the hemocoel and divided into abdominal heart and thoracic aorta (Miller, 1950; Rizki, 1978; Curtis et al., 1999; Rotstein and Paululat, 2016; Figure 1A). The walls of the heart consist of a layer of striated muscle cells helically oriented around the lumen, surrounded by pericardial cells. A layer of longitudinal muscle fibres, called ventral longitudinal muscle (VLM; sometimes LM), underlies the ventral surface of the adult heart (Jones, 1954; Chiang et al., 1990; Curtis et al., 1999; Meola et al., 2003; Lehmacher et al., 2012). A pair of AMs is present in each abdominal segment. Each AM is laterally attached to a discrete epidermal (exoskeletal) site and dorsally connects to the heart as a bundle of myofibers, with some fibres contacting the AMs in the adjacent segments along the surface of the heart (Figures 1B, B’). The number of described pairs of AMs in adult arthropods varies from 3 in the Dungeness crab (*Decapoda*) to 10 in the stick insect (*Phasmatodea*) and between 4 and 8 in *Diptera* and *Lepidoptera* (Table 1). Many studies of the circulatory system and associated AMs were conducted in evolutionarily successful holometabolous insects with separate larval and adult habitats (Truman, 2019), particularly species which either threaten human health or impact agriculture (Meola et al., 2003; Martins et al., 2011; Leódido et al., 2013; Table 1). Early physiological studies noted the absence of consistent link between AM contraction and heart beating rates, while severing of AMs could result into heart chamber collapse (Bullock and Horridge, 1965; and references herein). These data suggested a role of AMs controlling the hemolymph inflow through the ostia during diastole, not the heart beating rate (Rizki, 1978; Chiang et al., 1990; Ejaz and Lange; 2008; Glenn, et al., 2010). AMs in the moth *Hyalophora cecropia* and in *Locusta migratoria* were described as striated muscles for slow contraction, poor in mitochondria, therefore likely not in constant vigorous use (Sanger and McCann, 1968; Miller et al., 1979). An alternative scenario to AM contraction controlling the opening volume of the heart, was that AMs could be non-contractile muscles acting as elastic fibres. Finally, since adhering to the wall of the heart, AMs were also suggested to constitute a heart suspensory apparatus and, together with the VLMs, form a dorsal diaphragm partitioning the hemolymph into a dorsal sinus above the diaphragm and a ventral body cavity bathing internal organs (Jones, 1954; Miller et al., 1979; Bate, 1993; Miller, 1997). It is fair, however, to recognise that data scattering among many different arthropod species, coupled to the difficulty to manipulate AMs in living adults, has left many uncertainties about AMs properties and physiological functions. The existence of AMs in larvae of holometabolous insects, first depicted by Lowne (1890) and in detail by Jensen (1973) (Table 1) brought out new developmental issues. The discovery of thoracic alary-related muscles (TARMs) (Boukhatmi et al., 2014; Bataillé et al., 2015) raised new questions about the ontogeny, physiology and evolution of these muscles.

**TABLE 1:** Alary Muscles in different Arthropods. Arthropod species in which AMs were studied either in adults, or/and in larvae are listed by alphabetical order. Their characteristics and/or common name, order and (sub) family are indicated. Human diseases linked to insect species in bold are indicated. The reported numbers of AMs pairs are given. * refers to the discovery of TARMs in *Drosophila*.

Species name	Characteristics and/or common name	Order; (sub)family	Linked human diseases	AM pair number	References(s)
ADULTS					
***Aedes aegypti***	hematophagous mosquito	Diptera; Culicidae	chikungunya, dengue, Zika (virus)		Leódido, et al. (2013)
***Anopheles aquasalis***	hematophagous mosquito	Diptera; Anophelinae	malaria (Plasmodium vivax)		Barbosa da Silva et al., 2019
***Anopheles gambiae***	hematophagous mosquito	Diptera; Anophelinae	malaria (Plasmodium falciparum); lymphatic filariasis	6	Glenn et al. (2010)
*Baculum extradentatum*	walking stick	Phasmatodea; Clitumninae		10	Ejaz and Lange (2008)
*Carausius morosus*	common stick insect	Phasmatodea; Lonchodinae			Opoczynska-Sembratowa (1936), cited in Bullock and Horridge (1965)
***Culex quinquefasciatus***	hematophagous mosquito	Diptera; Culicidae	West Nile fever		Martins et al. (2011)
*Drosophila melanogaster*	fruit fly	Diptera; Drosophilidae		4	Curtis et al. (1999)
***Glossina morsitans***	tsetse (sleeping sickness) fly	Diptera; Glossinae	Trypanosomiasis (Trypanosoma brucei)	7	Meola et al. (2003)
*Manduca sexta*	tobacco hawk moth	Lepidoptera; Sphingidae			Dulcis, PhD thesis, Univ. Arizona, (2004)
*Marinogammarus marinus*	Amphipod; freshwater shrimp	Amphipoda; Gammaridae		9	Alexandrowicz, (1954)
***Panstrongylus megistus***	kissing bug	Hemiptera; Reduviidae	Chagas disease (Trypanoma cruzi)	8	Nogueira and de Souza (1991)
*Periplaneta americana*	american coakroach	Blattodea; Blattidae			Adams et al. (1973)
*Sphinx ligustri*	privet hawk moth	Lepidoptera; Sphingidae			Wasserthal and Wasserthal (1977)
*Toxorhynchites theobaldi*	phytophagous “elephant” mosquito	Diptera; Culicidae			Barbosa da Silva (2019)
***Rodniux prolixus***	kissing bug	Hemiptera; Reduviidae	Chagas disease (Trypanoma cruzi)	7	Chiang et al. (1990)
*Locusta migratoria*	Migratory locust	Orthoptera; Oedipodinae			Miller et al. (1979)
*Hyalophora cecropia*	Giant silk moth	Lepidoptera; Saturniidae			Sanger and McCann (1968)
*Caligo beltrao*	purple owl	Lepidoptera; Morphinae			Wasserthal and Wasserthal (1980)
LARVA/CATERPILLAR					
***Anopheles gambiae***	hematophagous mosquito	Diptera; Anophelinae	malaria (Plasmodium falciparum); lymphatic filariasis	9	League et al. (2015)
***Anopheles quadrimaculatus***	hematophagous “March” mosquito	Diptera; Anophelinae	malaria (Plasmodium falciparum)		Jones (1954)
*Bombyx Mori*	domestic silk moth	Lepidoptera; Bombicidae		8	Ai H and Kuwasawa (1995)
*Calliphora erythrocephala*	bow fly, house fly	Diptera; Calliphoridae		7	Lowne (1890); Jensen (1973)
*Drosophila melanogaster*	fruit fly	Diptera; Drosophilidae		7 + 3*	Labeau et al., (2009); Boukhatmi et al. (2014)
*Manduca sexta*	tobacco hawk moth	Lepidoptera; Sphingidae		7	Davis et al. (2001)

## Alary muscle founder cells

Robustness of the muscle pattern is crucial for an animal’s fitness and survival. Each body wall muscle, usually a large multinucleated syncytium in bilaterians, displays a species-specific morphology and capacity. In vertebrates, establishment of the skeletal muscle pattern - around 600 different muscles in humans - and myofiber differentiation are initiated and terminated in embryos, followed by muscle hypertrophy during the perinatal period. A pool of muscle stem cells (satellite cells) is maintained and required to maintain muscle homeostasis, growth and repair upon injury in adults (Buckingham and Montarras, 2008; Chang and Rudnicki, 2014). While specific molecular signatures have been identified for satellite cells associated to specific adult muscles types (Evano et al., 2020), developmental rules establishing stereotypical vertebrate muscle patterns and shapes only begin to be elucidated (Besse et al., 2020). In contrast, the molecular genetic basis of stereotypical muscle patterns has been highly investigated in the dipteran insect *Drosophila* (Bate, 1990; Bate, 1993; Dobi et al., 2015; Deng et al., 2017; Junion and Jagla, 2022).

**FIGURE 2:**
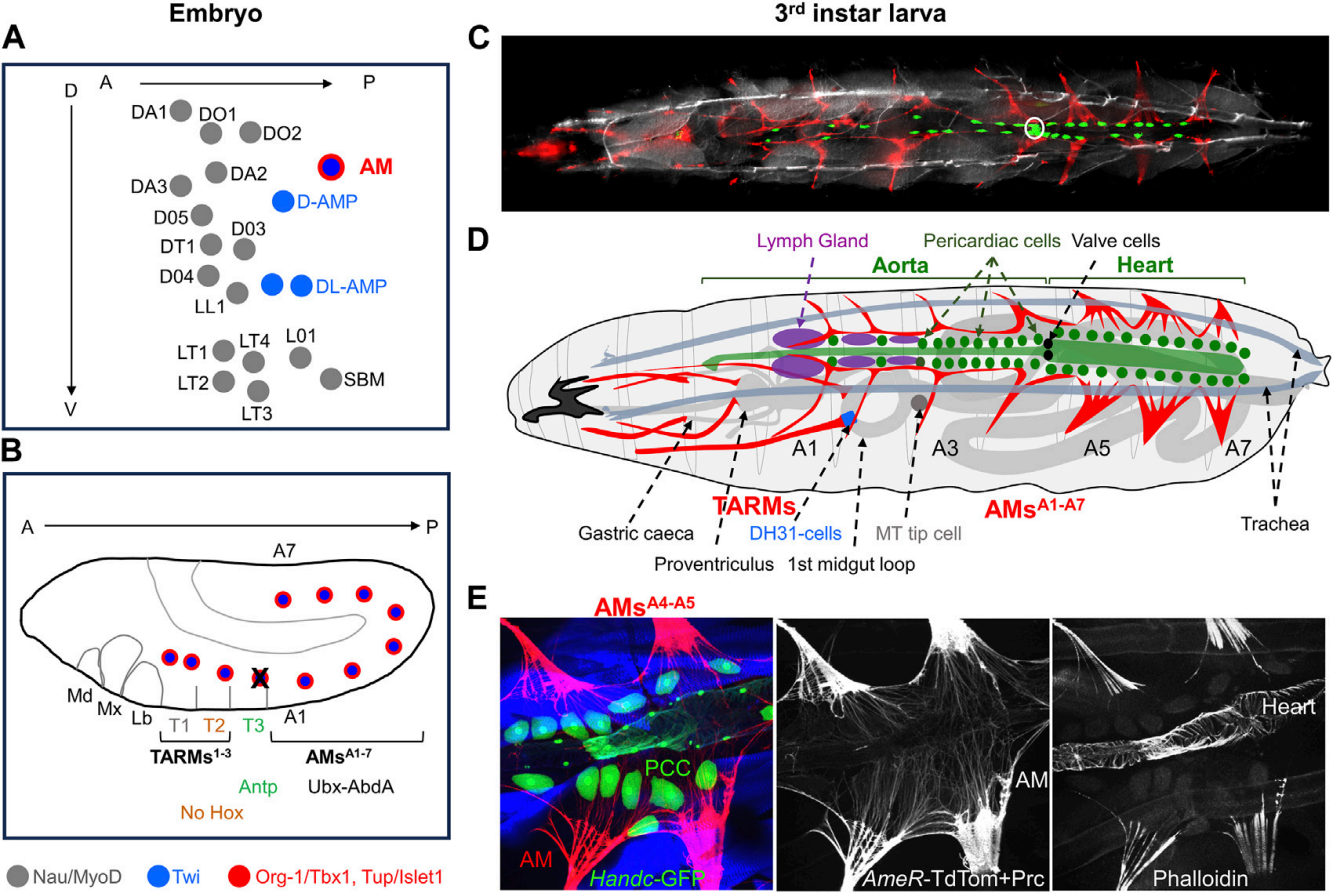
Larval AMs and TARMs. **(A)** Positions of muscle Founder Cells (FCs) at the origin of dorso/lateral skeletal muscles, AMs and Adult Muscle Precursors (AMPs) in an abdominal segment at embryonic stage 10 (Dobi et al. (2015)). Each FC is represented by a dot. FCs for skeletal muscles, designated by muscle initials and number (Bate, 1993), express Nau/MyoD (grey), AMPs express Twi (blue) and AM FCs express Twi plus Org-1/Tbx1 and Tup/Islet1 (red). **(B)** Schematic representation of Hox expression in AM FCs in a stage 11 embryo. Antp expression leads to AM apoptosis (black cross) in segment T3. **(C–E)** Third instar larvae. **(C)** Dorsal view of an intact *AME*_*R*_ *-Gal4; UAS-cd4-tdTomato, HandC-GFP* larva showing AMs and TARMs in red, cardiomyocytes, pericardial cells and valve cells (white circle) in green, and brightfield in grey to visualize the position of the dorsal tracheal trunks. **(D)** Schematic drawing of AMs and TARMs (adapted from Bataillé et al. (2020)). Abdominal AM^A1-A7^ and thoracic TARMs^1-3^ are drawn in red. AMs are internal to the dorsal trachea (grey blue), and connect dorsally to the ECM surrounding the pericardial cells and the dorsal vessel (green), and the Lymph Gland (purple). AM^A3^interacts with the tip cell of the anterior Malpighian tubule (MT). TARMs connect specific regions of the gut (light grey). **(E)** Detailed views of AM^A4-A5^ attachment to the heart viewed by confocal microscopy on a dissected *AME*_*R*_ *-cd4-tdTomato; HandC-GFP* larva stained with Phalloidin and Pericardin (Prc), showing AMs and Prc in red, pericardial cells (PCC), cardiomyocytes and valve cells in green, and skeletal muscles (SM) in blue. Median and right panels show AMs and Prc (red), and Phalloidin staining (blue), illustrating the ECM network prolongating the striated myofibrils and connecting AMs on either side of the heart and to the heart itself.

In holometabolous insects such as *Drosophila*, the embryo hatches into a motile larva. Metamorphosis marks the end of the larval growth period and initiation of the differentiation of adult tissues. During this process, most larval body wall muscles are histolysed and adult muscles form (Bate, 1993; Zirin et al., 2013). Thus, two successive muscle patterns underlie *Drosophila* larval and adult locomotion. The development of larval muscles, around 30 different muscles per hemisegment attached at precise positions to the larval exoskeleton, is initiated in early embryos (Bate, 1990). Each muscle is seeded by one founder myoblast, called Founder Cell (FC), able to fuse with fusion-competent myoblasts (Bate, 1990; Rushton et al., 1995). FCs are issued from asymmetric division of Progenitor Cells (PC) and each express a distinctive code of identity transcription factors (iTFs) which reflects both PCs positional values relative to the epidermis and developmental time (Frasch, 1999; Boukhatmi et al., 2012; Dobi et al., 2015; Figure 2A). iTF codes control muscle morphological identity, that is, each muscle-specific size, orientation and attachment sites to the epidermis via specialised tendon cells, and muscle/muscle matching at segment borders (Schweitzer et al., 2010; Dobi et al., 2015; Maartens and Brown, 2015; Carayon et al., 2020). *Drosophila* muscle iTFs include orthologues of mammalian myogenic TFs, such as MyoD/MRF (Muscle Regulatory Factor), Lbx, Islet1, Six and Tbx1 (de Joussineau et al., 2012; Buckingham and Rigby, 2014; Dubois et al., 2016). Nautilus (Nau), the *Drosophila* MRF ortholog, is expressed in all FCs before fusion (Michelson et al., 1990), before being restricted to and required in a small set of muscles (Balagopalan et al., 2001; Enriquez et al., 2012). A defined number of muscle PCs in abdominal segments divides into one FC and one adult muscle precursor (AMP). AMPs proliferate until metamorphosis, at which point most fuse together to *de novo* form adult muscles. AMPs are characterised by persistent expression of *Drosophila* Twist, a bHLH TF expressed early in all mesodermal cells (Bate et al., 1991; Figure 2A). Similar to vertebrates, a small number of AMPs is set aside to form a pool of satellite cells required for muscle repair in adults (Chaturvedi et al., 2017; Boukhatmi, 2021).

AMs are also seeded by embryonic FCs, the only FCs which co-express the T-box factor Org-1 (optomotor-blind-related-gene-1) and the LIM homeodomain TF Tailup (Tup) (Tao et al., 2007; Schaub et al., 2012; Boukhatmi et al., 2012; Boukhatmi et al., 2014; Figures 2A, B), the *Drosophila* orthologues of mammalians Tbx1 and Islet1, respectively (Thor and Thomas, 1997; Tao et al., 2007; Schaub et al., 2012). Unlike skeletal muscle FCs, AM FCs do not express Nau/MyoD, however, while retaining Twi expression, similar to AMPs (Boukhatmi et al., 2014; Schaub et al., 2015; Figure 2B). This unique Org-1^+^, Tup^+^, Twi^+^, Nau^−^ expression pattern together with the presence of AMs in both larvae and adults, is suggestive of a dual, embryonic and adult identity of AMs FCs.

## Development of AMs and thoracic alary-related muscles (TARMs)

At embryo hatching, the *Drosophila* dorsal vessel extends from the abdominal A7 segment forward to the thoracic T2/T3 segment boundary. Seven pairs of larval AMs, one per abdominal segment dorsally attach to the extracellular matrix (ECM) produced by the pericardial cells surrounding the layer of cardiomyocytes (Labeau et al., 2009; Drechsler et al., 2013; Boukhatmi et al., 2014; Figures 2C, D). That ECM plays a key role in attachment of embryonic AMs to the heart was illustrated by AMs detachment in mutant alleles of two ECM proteins, laminin B1 and Cg25C, one type IV collagen in *Drosophila* (Hollfelder et al., 2014). Another ECM constituent of the elastic connective tissue surrounding the embryonic *Drosophila* heart is Pericardin (Prc), a collagen IV-like protein (Chartier et al., 2002; Reinhardt et al., 2023). Upon Prc depletion, AMs come apart from the heart and the heart lumen collapses (Drechsler et al., 2013; Bataillé et al., 2020). Laterally, AMs attach to tendon cells situated at the intersegmental border. In their trajectory from the exoskeleton to the heart, AMs loop around main branches of the respiratory tracheal system. In addition different AMs contact other internal organs including the gonad and the fat body (Boukhatmi et al., 2014; Anllo et al., 2019). AM in segment A1 (AMs^A1^) connects the lymph gland (LG), the larval hematopoietic organ (Rizki, 1978; LaBeau et al., 2009; Bataillé et al., 2020; Figure 2D). An astonishing observation by Weavers and Skaer, 2013, was that the distal tip cells of developing anterior Malpighian tubules (MTs) successively adhere to AM^A5^, AM^A4^, and AM^A3^ during organogenesis, and that this sequential adhesion process is required for proper MT looping, and likely, effective hemolymph sampling. The AM/MT interaction was the first hint that AMs could establish, and be deformed by contacts with various tissues and be involved in positioning of internal organs in addition to the heart (Weavers and Skaer, 2013).

The observation of Org-1^+^/Tup^+^ expressing FCs in thoracic segments (Figure 2B) led to another astonishing discovery, the existence of three pairs of thoracic alary-related muscles (TARMs) connecting the exoskeleton to specific midgut regions (Boukhatmi et al., 2014; Figures 2C, D). Two TARMs, TARM^*^ and TARM^T1^, are seeded by FCs specified in thoracic segment T1 and attach to the proventriculus and to gastric caecae, respectively (Figures 2B, D). TARM^T2^ is seeded by a FC specified in T2 and connects to a precise position of the anterior midgut. The absence of Hox expression in TARM^T2^ is reminiscent of the situation in somatic muscles which led Roy et al. (1997), to propose that the T2 muscle pattern was the ‘ground state’. A TARM FC is specified in T3 but programmed cell death induced by Antp/HoxB7 activity interrupts TARM development in this segment (Bataillé et al., 2020; Figure 2B). AMs and TARMs attachment to the dorsal vessel and visceral organs, respectively, is also under Hox control. In Hox gain-of-function experiments, AMs form in thoracic segments (Labeau et al., 2009; Weavers and Skaer, 2013) instead of TARMs (Bataillé et al., 2015). Conversely, removal of posterior Hox (Ubx, Ultrabithorax) information in AM^A1^ and AM^A2^ leads to their transformation into TARM-like muscles connecting to the gut at the same position as TARM^T2^, suggesting that connection to endoderm is the default fate (Bataillé et al., 2015; Bataillé et al., 2020).

TARMs are the first described striated muscles connecting the exoskeleton to the gut in bilaterians. So far, TARMs have been documented neither in adult arthropods, nor in embryos of primitive ametabolous or hemimetabolous insects which hatch as a miniature version of the adult and do no not develop through a larval stage (Table 1). Investigating TARMs in a wide spectrum of arthropods could be the source of new discoveries.

## AMs and TARMS in larvae: Architectural and signalling functions?

Genetic analyses showed that embryonic AM and TARM development requires both *org-1* and *tup* functions (Boukhatmi et al., 2014). The design of AM/TARM-specific *org-1* and *tup*-expression reporter lines allowed in turn to specifically follow and ablate these muscles in larvae (Schaub et al., 2015; Bataillé et al., 2020). Morphological analyses confirmed that the shape of anterior and posterior AMs diversifies during larval development (Jensen, 1973). AMs^A5-A7^ adopt multi-fibre, fan-shaped connections to the heart (Figures 2C–E). AMs^A1-A3^ maintain a conspicuous tripolar “T” shape, with myofibres oriented ventro-dorsally from the exoskeleton to the aorta, then laterally along the aorta (Bataillé et al., 2020; Figure 2C and Supplementary Movie S1).

Targeted loss of AMs in larvae both leads to collapse of the cardiac vessel, recalling the proposed role in adults in AMs, and relieves topological constraints on curvature of the respiratory system. Loss of TARMs impairs positioning of the visceral mass. Therefore, AMs and TARMs collectively or individually maintain internal organs in proper position within the hemocoel (Bataillé et al., 2020). AMs/TARMs could also play signalling functions. TARM^T2^ attaches to the junction region of the anterior and acid-secreting portion of the larval midgut, where enteroendocrine cells expressing DH31 required for peristalsis are located (LaJeunesse et al., 2010). This attachment site and food transit reduction upon deletion of TARMs raise the possibility that TARMs could regulate endocrine functions. The lateral aspects of AM^A1^ run between the dorsal vessel and LG primary lobes and englobe the hematopoietic niche cells. Vesicles originating from AMs are detected in the aorta region situated between the LG lobes, suggesting that AMs could signal to the LG (Bataillé et al., 2020). Of note, the AM^A1^ pair is the only pair which does not detach in *laminin B1* mutants, suggesting a specific attachment mode (Hollfelder et al., 2014).

Undeniably, a most-peculiar feature of AMs and TARMs, revealed by live imaging of crawling larvae, is their extreme deformability/elasticity (Bataillé et al., 2020; Supplementary Movie S1). The multiple shapes adopted by AMs suggest that they could be passively deformed along each crawling stride cycle, during which internal organs move asynchronously with surrounding abdominal body wall (Heckscher et al., 2012). This deformability and asymmetric attachments to rigid and soft tissues, distinguishes AMs/TARMs from other striated muscles. In larvae, the sarcomeric AM fibres are prolonged by ECM rich fibres (Bataillé et al., 2020; Figure 2E). It was previously reported in *Calliphora* that systole causes considerable elongation of the elastic (dorsal) fibres from the alary muscles but only little elongation of the muscle fibres themselves (Jensen, 1973), an observation which remains to be investigated in depth. Whether AMs/TARMs express specific isoforms of Myosin heavy chain (MHC) and/or proteins of the sarcomere anchors (Kiehart et al., 1989; Kronert et al., 2012; Steinmetz et al., 2012; Cao and Jin, 2020; Murgia et al., 2021) to achieve peculiar deformability properties needs to be investigated, with biomaterials and biomedical perspectives.

## Trans-differentiation of AMs at metamorphosis

**FIGURE 3:**
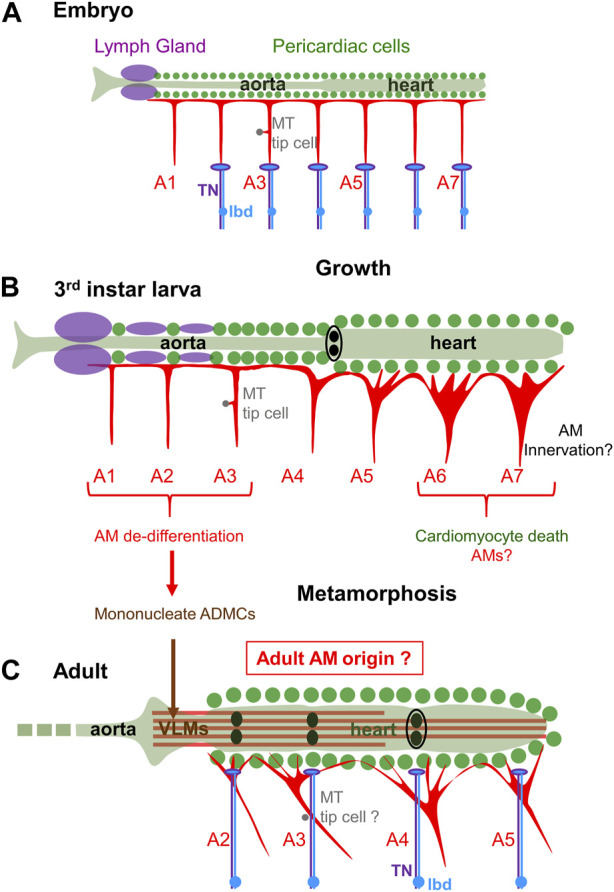
AMs fate at metamorphosis. **(A)** Schematic representation of the dorsal vessel and AMs in a late embryo. The 7 pairs of abdominal AMs^A1-A7^ display a similar T-shape. AMs^A2-7^ are innervated at their base by the TN motor neuron (violet) and dorsal dendrite of the peripheral lbd neuron (blue). **(B)** third instar larva: AMs have increased in size and diversified in morphology during larval growth. Innervation has not yet been described in detail. **(C)** After metamorphosis, 4 pairs of AMs, AMs^A2-5^ are found in adults. AM innervation is shifted to dorsal myocardium. Fragmentation of larval AMs^A1-3^ into mononucleate myoblasts (ADMCs) is followed by a new round of fusion into VLM. This trans-differentiation process and apoptosis of posterior AMs leaves uncertain the origin of adult AMs^A2-A5^.

Complete metamorphosis of holometabolous insects includes histolysis of abdominal larval body wall muscles and *de novo* formation of adult muscles. The presence of AMs both in larvae and adults of holometabolous insects therefore stands out as exception, and AM behavior during metamorphosis has intrigued entomologists for years (Jensen, 1973). The dorsal vessel is itself considerably restructured: the linear heart tube with one terminal wide-lumen heart chamber in larvae is converted into a linear four-chambered heart tube with three valves in adults (Rizki, 1978; Lehmacher et al., 2012; League et al., 2015; Rotstein and Paululat, 2016; Poliacikova et al., 2021; Meyer et al., 2023; Figures 3A–C). During this process « aortic » larval A1 to A4 myocytes are reprogrammed to acquire contractile properties while abdominal A5-A7 myocytes are eliminated by programmed cell death (Monier et al., 2005; Poliacikova et al., 2021). This results in forward shifting of the contractile heart from segments A5-A7 in embryos/larvae to A2-A5 in adults, the adult aorta being restricted to the thorax. Alongside, only a subset of AMs survive metamorphosis while VLMs are a new addition (Jensen, 1973; Curtis et al., 1999; Figures 3B, C). One hypothesis was VLMs could form by fusion of adult myoblasts with AM fragments (Curtis et al., 1999). AM fate and trans-differentaition into VLMs in *Drosophila* pupae has now been deciphered, using *in vivo* imaging, cell lineage and genetic analyses (Schaub et al., 2015; Schaub et al., 2019). These authors showed that larval AM^A1-A3^ undergo a lineage reprogramming process without proliferation. One first step is dedifferentiation and fragmentation of AM^A1-A3^ into mononucleated alary muscle derived cells (AMDCs), a step which involves JNK and Yorkie signalling. It is followed by a *de novo* round of fusion of AMDCs including recruitment of additional myoblasts from the pool of AMPs, and re-differentiation of *de novo* syncytia into VLMs (Figures 3B, C). Like AM FC specification, AM to VLM transdifferentiation is controlled by Org-1/Tbx1 and Tup/Islet1. It also requires Twi acting downstream of Org-1 (Schaub et al., 2015; Rose et al., 2022). Further dissection of this naturally occurring transdifferentiation process will likely bring more information into mechanisms of cellular reprogramming during ontogeny and tissue regeneration. Trans-differentiation of larval AM^A1-A3^, together with removal of posterior AMs and maintenance of AM^A4^ connection to the posterior cardiac valve region (Jensen, 1973; Monier et al., 2005; Meyer et al., 2023) leaves unclear, however, how adults AM^A2-A5^ are remodelled during metamorphosis (Figures 3B, C). More broadly, how reprogramming of larval aorta into contractile cardiomyocytes, transdifferentiation of specific cardiomyocytes into valve cells, transdifferentiation of anterior AMs into VLM and connection of AM^A5^-AM^A7^ to the adult heart is coordinated during metamorphosis to generate a functional adult dorsal vessel, remains a fascinating question.

Both skeletal muscles, AMs and TARMs, considerably enlarge during *Drosophila* larval development to accommodate increasing body volume. For skeletal muscles, fusion of a FC with a defined numbers of FCMs in embryos sets the number of nuclei specific to each muscle. Muscle size increase is accompanied by an increased size, not number, of nuclei with endoreplication stepping up the DNA content within each nucleus. Nuclear scaling, i.e., maintaining a stable scaling of DNA content with muscle size, relies upon muscle individual increase of nuclear ploidy (Demontis and Perrimon, 2009; Windner et al., 2019). Polyploidization of cardiac and pericardial cells (Jensen, 1973; Chakraborty et al., 2023) also accompanies the increase in length of the *Drosophila* heart. The number of nuclei per AM/TARM in L3 larvae is between 4 and 6 (Bataillé et al., 2020), similar to the number at embryo hatching (Boukhatmi et al., 2014; Rose et al., 2022), suggesting as for skeletal muscles the absence of nuclear divisions during larval development. Yet, the ability of at least a subset of multinucleate AMs to dedifferentiate into mononucleated alary muscle derived cells (AMDCs) and fuse with additional myoblasts during VLM formation (Schaub et al., 2015; 2019; Figure 3B) suggests that these AM nuclei are diploid at the onset of metamorphosis, something which remains to be ascertained. How to reconcile AM nuclear diploidy and AM growth could then be addressed. More globally, the dual embryonic and adult identity of AM nuclei suggests specific properties. Localized interactions of AMs with other tissues, such as the LG or the MT tip cell further raises the question of whether some AM nuclei are specialized to regulate these local interactions.

## AMs dual innervation?

Innervation of the heart in the control of heart-beating and hemolymph flux has been investigated in various insects and crustaceans (Alexandrowicz, 1954; Bullock, T. H. and Horridge, 1965; Miller and Usherwood, 1971; Jones, 1977; Miller et al., 1979). Several questions related to AMs role(s) in heart control needed to be addressed: whether AMs were innervated, independent of heart, and by which type of neurons (Wasserthal and Wasserthal, 1977; Carr and Taghert, 1988; Chiang et al., 1990; Ai and Kuwasawa, 1995; Miller, 1997).

The present view is that adult AMs, or a subset, are innervated by the dorsal branch of a segmentally repeated nerve (alternately called dorsal nerve or transverse nerve (TN)), with neuron-AMs junctions located at their junction to the myocardium (Wasserthal and Wasserthal, 1977; Carr and Taghert, 1988; Chiang et al., 1990; Ai and Kuwasawa, 1995; Miller, 1997; Dulcis and Levine, 2003; Meola et al., 2003). Dorsal projections of the TN have been observed to fasciculate with a peripheral bipolar neuron (lbd), also designated as BpN, BpN2 or L1 (Wasserthal and Wasserthal, 1980 *;* Bodmer and Jan, 1987; Miller, 1997; Dulcis and Levine, 2003; Williams and Shepherd, 1999; Ejaz and Lange, 2008; Figure 3C). An FMRFamide neuromediator were previously co-localised to the dorsal unpaired median (DUM) heart-1a neuron which projects to the heart and AMs in locusts (Stevenson and Pflüger, 1994; Lange et al., 2009). Glutamate immunostaining was also detected in the *Drosophila* abdominal heart (Dulcis and Levine, 2003). Innervation of adult AMs could thus comprise excitatory and neurosecretory innervation. It remains to separate out which neuron (terminals) are active on the adult AMs and on the heart itself, and the specific roles of the peptidergic and glutamatergic innervation (Miller, 1997; Dulcis and Levine, 2003).

In larvae, heart position and lumen opening are constrained by AMs (Bataillé et al., 2020), but not heart-beating activity which is myogenic. However, Gorczyca et al. (1994), found that AMs were already innervated by the TN in late embryos, at their base, not dorsal attachment to the heart, unlike proposed in adults (Figure 3A). The embryonic TN extends dorsally from the CNS and reaches the cell body of the lbd neuron along the segmental boundary after branching off to innervate one skeletal muscle, the ventral transverse muscle 1 (VT1) (Gorczyca et al., 1994; Macleod et al., 2003; Landgraf and Thor, 2006). The dorsal dendrite of the lbd neuron travels with the TN to the base of the AM (Gorczyca et al., 1994; Figure 3A). Correlatively, the structure of the embryonic AM neuromuscular junction displays both features of excitatory motoneurons with the postsynaptic marker, Disc Large (DLG), accumulation around boutons (Wang X. et al., 2022) and thick neuritic endings diagnostic of the tip of sensory neurons (Gorczyca et al., 1994). Thus, both embryonic and adult data suggest a dual AM innervation, motor and peripheral, deviating from the rule of insect skeletal muscles solely innervated by motoneurons (Kohsaka et al., 2012).

**FIGURE 4:**
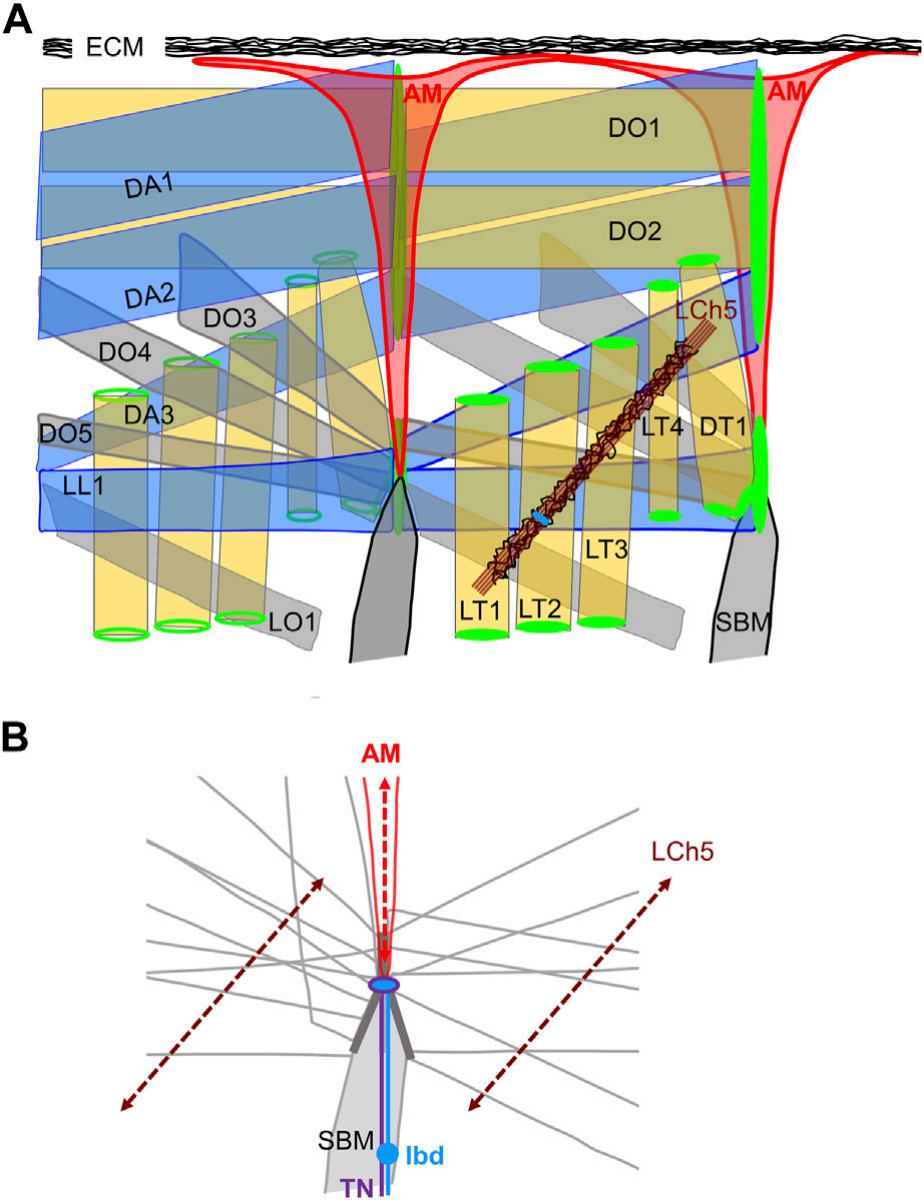
The dorso-lateral muscles. **(A)** Two consecutive segments are shown. Left segment, internal view; right segment, external view. AMs are the internal-most muscles. Each muscle is designated by its abbreviated name (Bate, 1993). The dorso-lateral muscle attachment sites at each segmental border are drawn in green, the lateral pentascolopidial chordotonal organs (LCh5) in brown, with neuron cell bodies in blue. Pericardin-rich ECM is drawn in black. **(B)** The contours of muscles attached to the lateral intersegmental epidermal attachment site are drawn, illustrating muscle-muscle matching interfaces (dark grey) and the nodal attachment site of AMs. The dorsal projections of the TN (violet) and LBD (blue) neurons reach the base of AMs. The stretching axes of the LCh5 ligament cells and the AMs are schematised by dotted double arrows.

The question of whether AM function(s) is active or passive in relation to heart beat and possibly in coordination with the animal motion was introduced by Alexandrowicz (1954). In mammals, feedback proprioceptive information from muscle to the CNS is provided by sensory innervation of intrafusal muscle fibres (Barker and Chin, 1961; Kröger and Watkins, 2021; Dimitriou, 2022). In the *Drosophila* larva, proprioceptive information is provided by surface touch neurons and neurons of stretch-receptive, chordotonal organs (ChO). Neither are directly connected to larval skeletal muscles (Ghysen and Dambly-Chaudiere, 1989; Brewster and Bodmer, 1995; Hassan et al., 2019). The base of AMs, the site of neuro-AM junction prior to metamorphosis, superimposes a nodal epidermal attachment site of many skeletal muscles (Labeau et al., 2009; Boukhatmi et al., 2014; Bataillé et al., 2020; Figure 4). This location raises the admittedly speculative possibility that the lbd neuron could sense AM stretching during locomotory contraction and relaxation waves, and feed-back information to the TN neuron. Intrasegmental contractions of lateral muscles are sensed by the lateral LCh5 chordotonal organs (Figure 4A) (Caldwell et al., 2003; Klein et al., 2010; Hassan et al., 2019). The stretching axes of AMs and LCh5 could possibly form a proprioceptive grid (Figure 4B). Prior to speculating further, many functional data are needed. It remains unknown whether the TN controls AMs contraction, and whether the neuronal input from the ldb is neurosecretory and/or carries a sensory feed-back function.

The lbd is one peripheral neuron which persists from larval to adult (Williams and Shepherd, 1999). During metamorphosis, TN dorsal arborizations ramify extensively along cardiac chambers and associated AM strands (Dulcis and Levine, 2003), such that AM innervation seems to be shifted from its base in larvae, to strands reaching pericardial ECM in adults (Figures 3A–C). Whether the lbd and TN neurons are part of the same neuronal circuit(s) in larvae and adults also remains to be deciphered.

## Ancestral origin and evolution of AMs; the Tbx1-Islet1 (Twi) network

An unusual feature of AMs/TARMs which distinguishes them form cardiac, skeletal and visceral muscles is their asymmetric attachment, to the exoskeleton on the one hand, and either the cardiac or the visceral mesoderm, on the other. In mammals, the muscle diaphragm which separates lung and heart from visceral organs is also an asymmetric striated muscle. Its peculiar C-shape results from insertion of lateral muscular fibres into bones, either ribs or vertebrae, while central fibres are organised around a sheet of fibrous tissue, the central tendon which surrounds the esophageal hiatus (Merrell and Kardon, 2013). Although highly speculative, whether the mammalian diaphragm and the insect AMs/TARMs and VLM could represent two specific adaptations of an ancestral demarcation between dorsal circulatory and respiratory, and ventral visceral organs, is one possibility. In primates, facial subcutaneous muscles display asymmetric attachment, into the skin on one side, and to facial bones or other muscles, on the other (Heude et al., 2018; Ziermann et al., 2018). Some of these muscles derive from the cardiopharyngeal mesoderm, also at the origin of the esophagus striated muscle (ESM) which forms in the absence of a primary skeletal muscle scaffold. Tbx1 and Islet1 are required cell-autonomously for specification of ESM progenitors, Tbx1 acting genetically upstream of Islet1 (Gopalakrishnan et al., 2015; Comai et al., 2019). More broadly, Tbx1 and Islet1 are major conserved actors in the genetic program controlling pharyngeal muscle development in chordates while Twi is involved in formation and regeneration of extraocular muscles (Nathan et al., 2008; Sambasivan et al., 2011; Zhao et al., 2020; Whitman et al., 2022). In *Drosophila,* the Tbx1/Islet1 genetic hierarchy selectively controls AM/TARM development and, together with Twi, AM into VLM trans-differentiation (Boukhatmi et al., 2014; Schaub et al., 2015; Rose et al., 2022). Whether the Tbx1/Islet1 hierarchy has been recruited during evolution for diversification and specific adaptations of striated muscles is an open question. Extant cnidarians display myoepithelial cells that are fully integrated into the ectodermal and endodermal epithelial tissues. These specialized cells which contain interconnected contractile basal extensions play equivalent roles to muscle layers (Leclère and Röttinger, 2017). In medusae, locomotion is achieved by the rhythmic pulsation of circular sheets of epithelial striated muscles located around the bell margins and lining the subumbrellar surface. Their contractions are counteracted by the elastic properties and antagonistic force of the ECM (mesoglea) (Leclère and Röttinger, 2017; Wang Y. et al., 2022). It would certainly be rewarding to investigate whether the Tbx1-Islet1 (Twi) regulatory hierarchy operates in muscles of cnidarians and/or other diploblastic animals and contributes to specifying specific mechanical/elastic properties such as those found in AMs/TARMs.

## Concluding remarks

AMs and TARMs are multinucleate striated muscles connecting the exoskeleton to multiple internal organs in insects. Several critical questions remain unanswered, among which the modes and roles of AM innervation, their mechanical properties, and their evolutionary origin. Further characterization of these still mysterious muscles is expected to bring original insight into the processes of anatomical, and physiological diversification of striated muscles throughout evolution.

## Supplementary material

SUPPLEMENTARY VIDEO S1 AMs deformations during larval crawling. Crawling 3^rd^ instar larva. AMs and heart cells nuclei -cardiac, pericardial and valve- are visualised through expression of *AME*_*R*_ *-Gal4, UAS-cd4-tdGFP* and *HandC-GFP,* respectively.

## Abbreviations

AM, Alary muscle; AM^A1^, Alary muscle from Abdominal segment 1; AMDC, Alary muscle derived cell; AMP, Adult muscle precursor; ChO, Chordotonal organ; CNS, Central nervous system; DH31, Diuretic hormone 31; ECM, Extracellular matrix; ESM, Esophagus striated muscle; FC, Founder cell; FCM, Fusion-competent myoblast; (i)TF, (identity) Transcription factor; L3 larva, 3rd instar larva; Lbd, peripheral bipolar neuron; LCh5, Lateral pentascolopidial chordotonal organ; LG, Lymph gland; MT, Malpighian tubule; PC, Progenitor Cell; PCC, Pericardial cell; SM, Skeletal/somatic muscle; TARM, thoracic alary-related muscle; TARM^T1^, Thoracic AM-related muscle from the Thoracic segment 1; TN, Transverse nerve; VLM, Ventral longitudinal muscle.
